# MartiTracks: A Geometrical Approach for Identifying Geographical Patterns of Distribution

**DOI:** 10.1371/journal.pone.0018460

**Published:** 2011-04-12

**Authors:** Susy Echeverría-Londoño, Daniel Rafael Miranda-Esquivel

**Affiliations:** Laboratorio de Sistemática y Biogeografía, Escuela de Biología, Universidad Industrial de Santander, Bucaramanga, Santander, Colombia; American Museum of Natural History, United States of America

## Abstract

Panbiogeography represents an evolutionary approach to biogeography, using rational cost-efficient methods to reduce initial complexity to locality data, and depict general distribution patterns. However, few quantitative, and automated panbiogeographic methods exist. In this study, we propose a new algorithm, within a quantitative, geometrical framework, to perform panbiogeographical analyses as an alternative to more traditional methods. The algorithm first calculates a minimum spanning tree, an individual track for each species in a panbiogeographic context. Then the spatial congruence among segments of the minimum spanning trees is calculated using five congruence parameters, producing a general distribution pattern. In addition, the algorithm removes the ambiguity, and subjectivity often present in a manual panbiogeographic analysis. Results from two empirical examples using 61 species of the genus *Bomarea* (2340 records), and 1031 genera of both plants and animals (100118 records) distributed across the Northern Andes, demonstrated that a geometrical approach to panbiogeography is a feasible quantitative method to determine general distribution patterns for taxa, reducing complexity, and the time needed for managing large data sets.

## Introduction

The geographic distribution of species has been considered an important source for documenting and conserving biodiversity [1]. Given the exponential growth of distributional data [2], [3], the necessity for procedures and bioinformatics tools to facilitate data management, reduce data complexity, and find general patterns from distributional point records has increased.

In this context, different biogeographic approaches make use of tools, to manage and analyze these kind of data. Within these approaches panbiogeography is considered an important tool for the primary management of distributional data [4], because it focuses on the spatial or geographical component, as a fundamental precondition to any analysis of the patterns and processes of evolutionary change [5]–[7]. This evolutionary approach to biogeography was developed by Croizat [8]–[10], as a response to Darwin's biogeographic ideas on means of dispersal in geographic distribution [11].

Panbiogeography delimits distributional patterns for multiple species and is known as track analysis. This method is based on three graphic elements: individual tracks, generalized tracks, and nodes [5], [7], [12], [13]. An individual track is made up of lines drawn on a map, on which different localities or distribution points of a particular taxon are connected, such that the sum of the segment lengths connecting all distribution points is the smallest possible. In graph theory, an individual track is a minimum spanning tree (hereafter MST) [5], [14], [15]. Generalized tracks, or standard tracks, are lines on a map resulting from overlapping individual tracks, as such, they are considered repetitive patterns summarizing the distributions of diverse individual taxa [16]. These patterns reflect an ancestral biota that has been fragmented by tectonic or climatic events [17]. Finally, nodes are areas where two or more generalized tracks overlap. These are complex areas or tectonic and biotic convergence zones [12], [14], [15], [17]. Thus, these three elements (individual tracks, generalized tracks, and nodes) define the main steps of track analysis [14]. First, two or more individual tracks are calculated from geographic locality records, then generalized tracks are delimited through geographic congruence of individual tracks, and finally, nodes are identified as the intersection area(s) between generalized tracks.

Different approaches exist within panbiogeographic methods. For example, Croizat's manual reconstruction [9], [10], Page's spanning graphs [15], Craw's track compatibility [18], and PAE (“Parsimony Analysis of Endemicity”) [5], [19]–[22]. Nevertheless, there are few quantitative and automated approaches for mapping generalized tracks (e.g. Craw's compatibility track analysis [18], [23]) with software implementations.

Considering that individual and generalized tracks are lines in a geometrical context, and congruence of individual tracks is a geometric property, in this study, we describe new software, named MartiTracks, based on a new algorithm to perform a panbiogeographic track analysis using a geometrical approach. The algorithm includes geometric functions and processes, which makes this approach a feasible quantitative alternative to the traditional track analysis. Finally, this approach is a unique and useful technique to capture distributional patterns or structures in studies employing spatial data.

## Results

### The general framework

**Figure 1:**
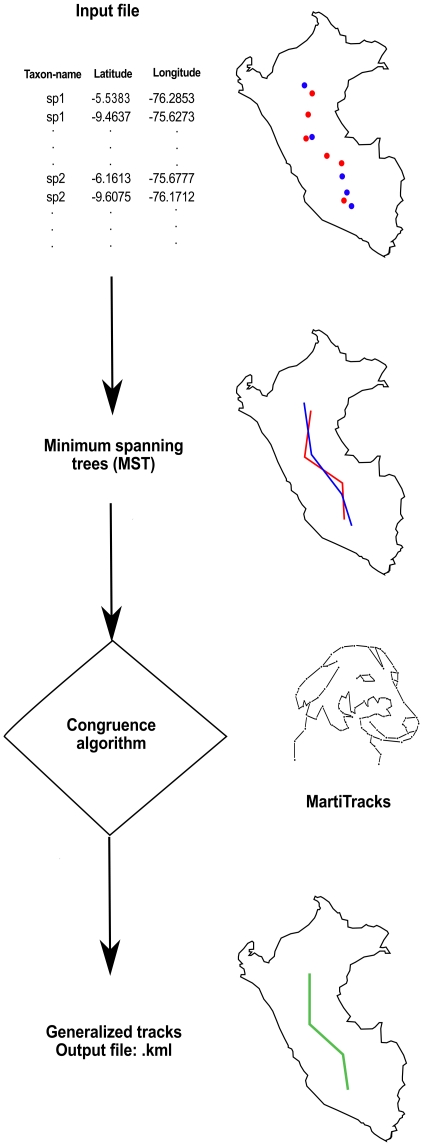
MartiTracks' framework. The user specifies an input file containing species distributional data (latitude-longitude). Then, these geographic points are used to calculate a minimum spanning tree (MST) for each species. Finally, the MSTs are analyzed by the congruence algorithm in order to delimit general patterns of distribution. The output is a KML file.

For a new MartiTracks project, distribution point records (latitude and longitude data) of a particular set of taxa must be compiled. A typical MartiTracks input file consists of a text file, which has the following structure: taxon-name, latitude, and longitude data. These data points are utilized to build an individual track for each species. The spatial congruence of the individual tracks is then evaluated through the congruence algorithm in order to determine whether there are generalized tracks representing the general patterns of distribution. Finally, the individual tracks of each species and the generalized patterns of distribution are represented in a KML (Keyhole Markup Language) file that can be visualized using any Geographic Information System (GIS) program such as GoogleEarth, or Qgis (Figure 1).

### First step: Minimum spanning trees (MST)

In the same way as most of panbiogeographic software, for example, Croizat [23], or Trazos2004 [24], MartiTracks initially creates an MST, representing an individual track. When two or more points are found at the same place, or are close enough to be considered the same sampling point, these points are reduced to a single point, using a minimum Euclidean distance parameter that we called cut value. Therefore, this parameter reduces initial redundancy in the data sets, speeding up the calculation of MSTs.

### Second step: Spatial congruence among species

#### Spatial congruence between two MSTs

**Figure 2:**
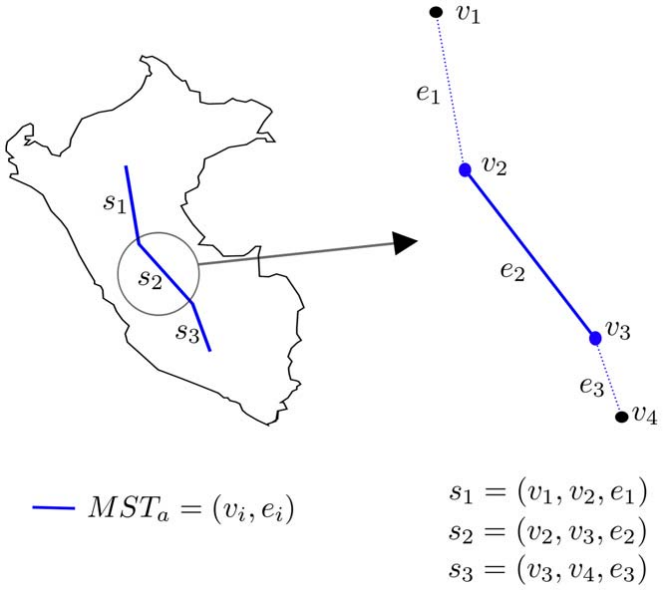
Basic units of congruence. Segments of the MSTs are the basic units of congruence between two species. Each segment 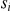 belonging to the 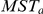 is defined as an edge 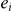 that connects two endpoint vertices 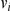.

Once the individual tracks are defined, the panbiogeographic method determines the spatial congruence of the individual tracks in order to delimit generalized tracks representing general patterns of distribution. The geometrical approach of MartiTracks considers each MST' segment or edge as the basic unit of congruence between two species. Thus, given an individual track or MST as 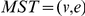 involving a set 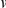 of vertices together with a set 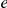 of edges, a segment 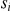 belonging to 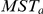 is defined as the edge 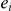 connecting two endpoint vertices 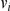 (Figure 2).

**Figure 3:**
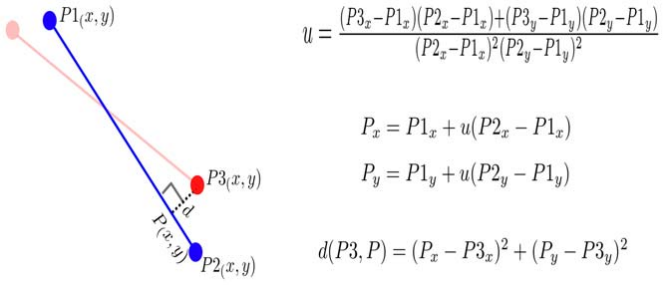
Distance from a point to a segment. The distance from point (P3) to segment (P1–P2) is calculated by the distance 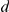 between point P3 and the intersecting point (P), resulting from the perpendicular extension of P3 towards segment (P1–P2).

The core of MartiTracks' geometrical approach is the function that calculates the shortest distance from a point to a segment. This function was developed by Paul Bourke and can be found at http://local.wasp.uwa.edu.au/pbourke/geometry/pointline/. Given segment (P1–P2) and point P3 (Figure 3), the distance 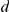, from point P3 to segment P1–P2 is defined as the distance between point P3 and the intersecting point P, resulting from the perpendicular extension of P3 towards segment P1–P2. If there is no intersecting point from the perpendicular extension of P3, the function will take the shortest distance from point P3 to either endpoint of segment P1–P2.

**Figure 4:**
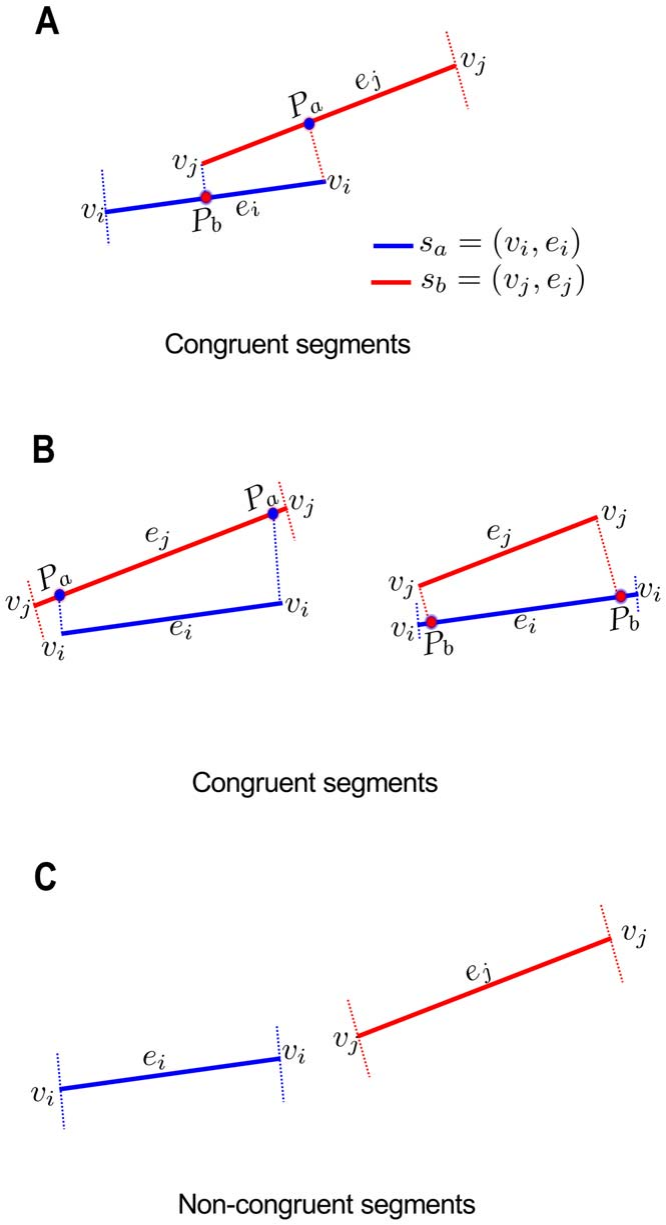
Conditions of congruence. MartiTracks considers two segments 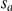, and 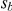 as congruent, **A**. if any of the vertices 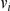 has an intersecting point 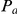 on edge 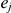, or if any of the vertices 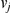 has an intersecting point 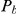 on edge 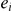 **B**. if both vertices 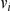 intersect on edge 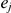, or if both vertices 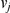 intersect on edge 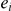. **C**. There is no congruence between segments if there is no intersecting points between them.

Given two segments 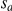, and 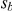 belonging to species 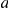 and 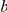, respectively, we consider that these two segments are congruent if any of the vertices 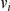 in segment 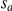 has an intersecting point 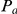 on 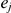, or if any of the vertices 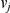 in segment 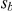 has an intersecting point 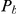 on 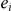 (Figure 4A); or if both vertices 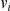 in segment 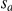 intersect on 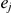, or if both vertices 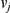 in segment 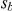 intersect on 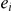 (Figure 4B). If there are no intersecting points 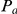 or 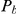 on edges 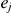, and 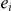 respectively, then segments 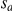, and 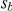 are not congruent (Figure 4C).

As congruence also depends on the Euclidean distances between segments and points, the maximum and minimum distances between segments are calculated in order to define two decision rules of congruence. Using these rules, two segments are congruent if the minimum, and maximum distances between segments do not exceed the predefined limits.

**Figure 5:**
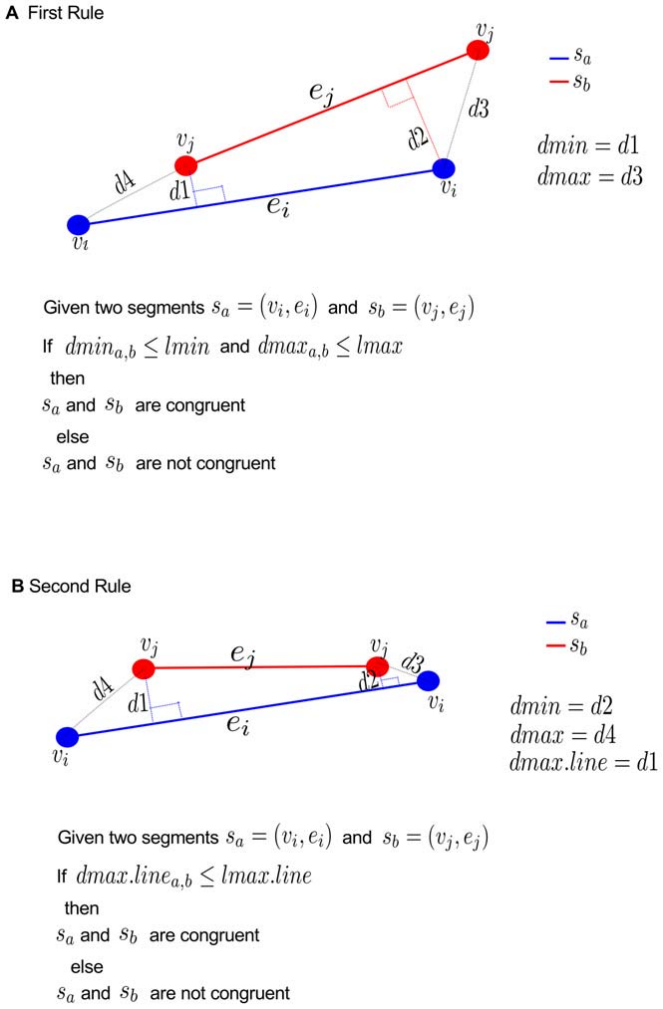
Decision rules of congruence. MartiTracks takes the minimum, and the maximum distances between segments to define the decision rules of congruence. Given two segments 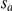, and 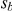 belonging to species 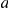 and 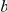, respectively; two segments are congruent if: **A**. the first condition of congruence is fulfilled (see Figure 4A), and if (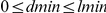) and (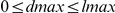) are true. **B**. If both 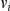 have intersecting points on 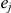 or if both 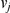 have intersecting points on 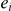 (see Figure 4B); and if (0
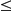
dmax.line
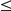
lmax.line) is true.

For the first rule, given 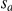, and 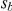 belonging to the species 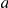 and 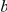, respectively, where 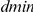 is the minimum distance, 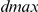 the maximum distance, 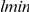 the boundary of the minimum distance, and 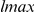 the boundary of the maximum distance. Two segments are congruent, if the first congruence condition is fulfilled (Figure 4A), and if (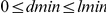) and (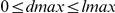) are true (see Figure 5A).

The second rule is defined by the maximum distance within the spatial range. Given two segments 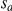, and 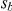 belonging to species 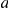 and 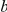, respectively, where dmax.line is the maximum distance within of the line segment, and lmax.line the boundary of the maximum distance within of the line segment. The two segments are congruent, if both 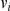 in segment 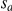 have intersecting points on 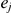 or if both 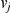 in segment 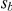 have intersecting points on 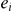 (Figure 4B), and if (0
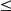
dmax.line
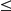
lmax.line) is true (see Figure 5B).

Finally, if two segments are found to be congruent, their points will be connected through a new MST. Then, each segment of species 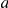 is compared to all other segments of species 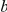 until the whole MST of species 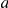 has been compared. The same procedure is carried out from species 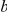 to 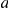. If the congruence between two species is null, no tracks or new MSTs will be created.

#### Spatial congruence among MSTs

**Figure 6:**
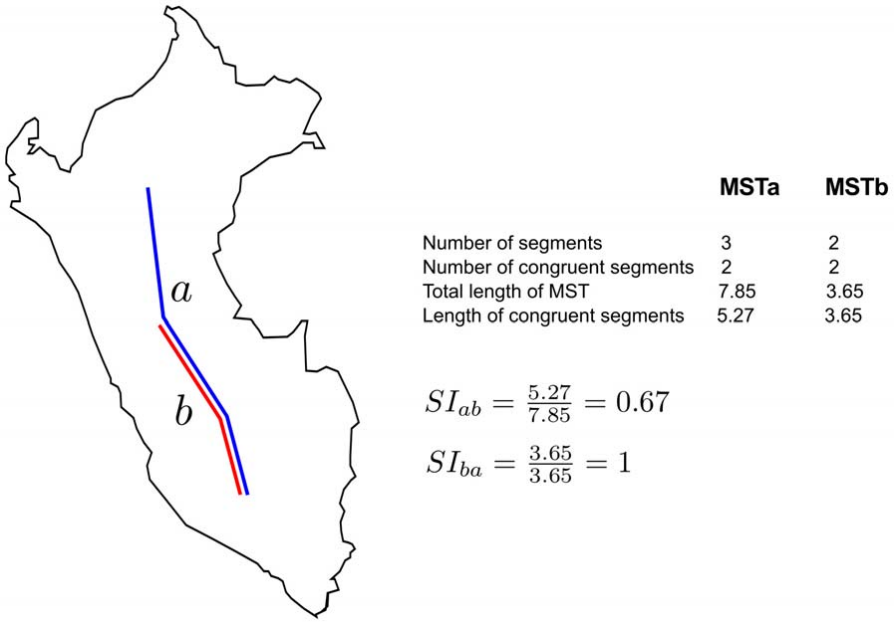
Similarity index (SI). MartiTracks calculates the similarity among tracks through a similarity index (SI). Given two tracks 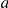, 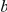 (either individual, or generalized tracks), the similarity index 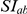 is the length of the congruent segments from 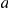 to 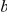 divided by the total length of the 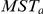. In the same way the similarity index 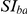 is the length of the congruent segments from 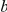 to 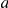 divided by the total length of the 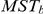.

Therefore, the spatial congruence among MSTs is the criterion to define whether a generalized track exists; if a species is not congruent with the remaining species, no generalized tracks are generated. Once, all species are compared and some levels of congruence are detected, a generalized track is created. When the analysis is complete, some repeated tracks may result, which can be reduced to a unique solution by means of a similarity index (SI). This index (SI) measures the similarity between tracks (either individual, or generalized tracks), and depending on a pre-established threshold, determines whether two tracks can be considered as the same element or not. Given two MSTs 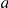 and 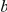 (Figure 6), the similarity index between them is calculated taking into account the length of their congruent segments, and the total length of 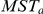, and 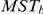.

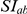
 = length of congruent segments 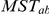/total length of 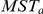

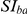
 = length of congruent segments 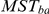/total length of 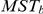.

It is important to emphasize that this is an asymmetrical index, due to its dependence on the length of the MSTs. Thus, 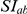 is different to 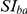, because 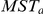 is longer than 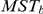 (Figure 6). Given 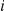 as the higher value between 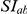, and 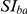; and min-SI as the predefined threshold value of SI, if (i
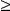
min-SI) the geographical points of the MST of species 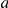, and 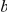 are joined, and they become part of the same MST.

Finally, the parameters cut value, lmin, lmax, lmax.line, and min-SI can be predefined according to the user's required level of congruence. It is important to consider that the value of each parameter of congruence depends on the value of the other parameters. Similarly, there is a constraining rule for these values, hence the cut value
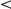
lmin
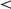
lmax
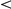
lmax.line.

### Empirical analyses

#### Panbiogeographical analysis of the genus *Bomarea* (Alstroemeriaceae)

**Figure 7:**
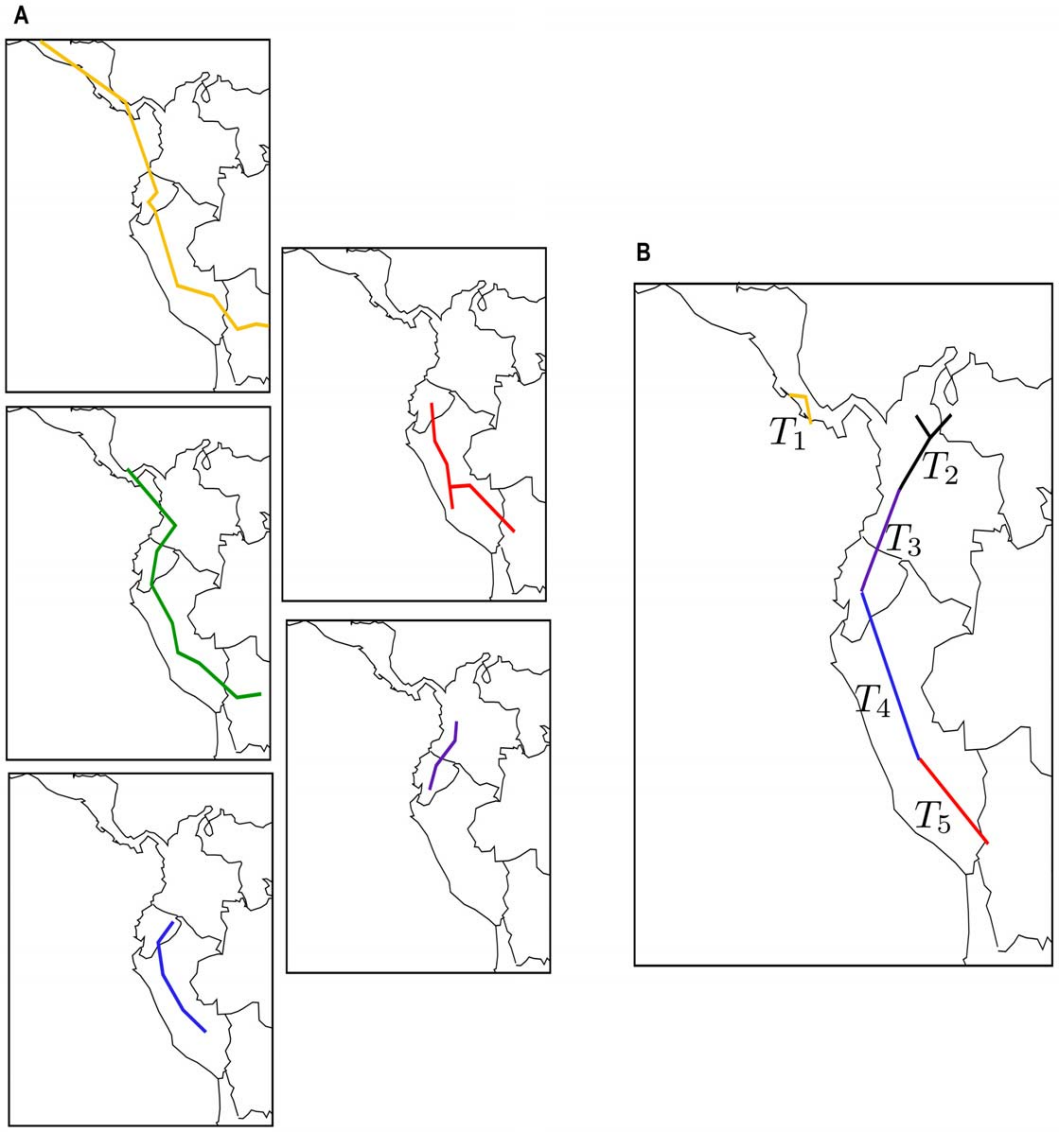
Panbiogeographical analysis of the genus *Bomarea* (Alstroemeriaceae). **A**. The five generalized tracks obtained with the following parameters: cut value = 2, lmin = 2.5, lmax = 3. lmax.line = 4, and min-SI = 0.8 in MartiTracks. **B**. Generalized tracks from Alzate *et al.* [25].

An empirical analysis was conducted with 2340 records belonging to 61 species of the genus *Bomarea*, obtained from the Global Biodiversity Information Facility GBIF ( http://www.gbif.org/datasets/resources/ 24/07/2010). We used three different sets of parameters values in order to calculate the general distributional patterns of *Bomarea* with different levels of congruence. The generalized tracks obtained by one of the sets are shown in Figure 7A.

To run the program, we used a PC-compatible computer with an Intel Core 2 Quad Q6600 at 2.40 GHz and 4 GB of RAM, running Ubuntu 9.04 64 bits. The panbiogeographical analyses of the genus required 30 to 60 seconds.

These results were compared to the results of a previous panbiogeographic work on *Bomarea*, using a traditional panbiogeographic analysis by Alzate *et al.* [25]. In contrast to our analysis, Alzate *et al.* used 2205 records belonging to 101 species of the genus *Bomarea*. Although there is a difference between the number of species evaluated in both analyses, similar patterns of distribution were found (Figure 7B).

#### Panbiogeographical analysis from the Northern Andes

We analyzed 100118 georeferenced records belonging to 1031 genera of plants and animals, distributed across the Northern Andes, in order to evaluate MartiTracks efficiency with large data sets. This data set was obtained from the Global Biodiversity Information Facility GBIF (http://www.gbif.org/datasets/resources/26/06/2009), and was not filtered for errors in distributions or taxonomy; therefore, mimicking an exploratory analysis to evaluate a very large data set. Four parameter sets were employed to visualize general patterns of distribution with different levels of congruence.

**Figure 8:**
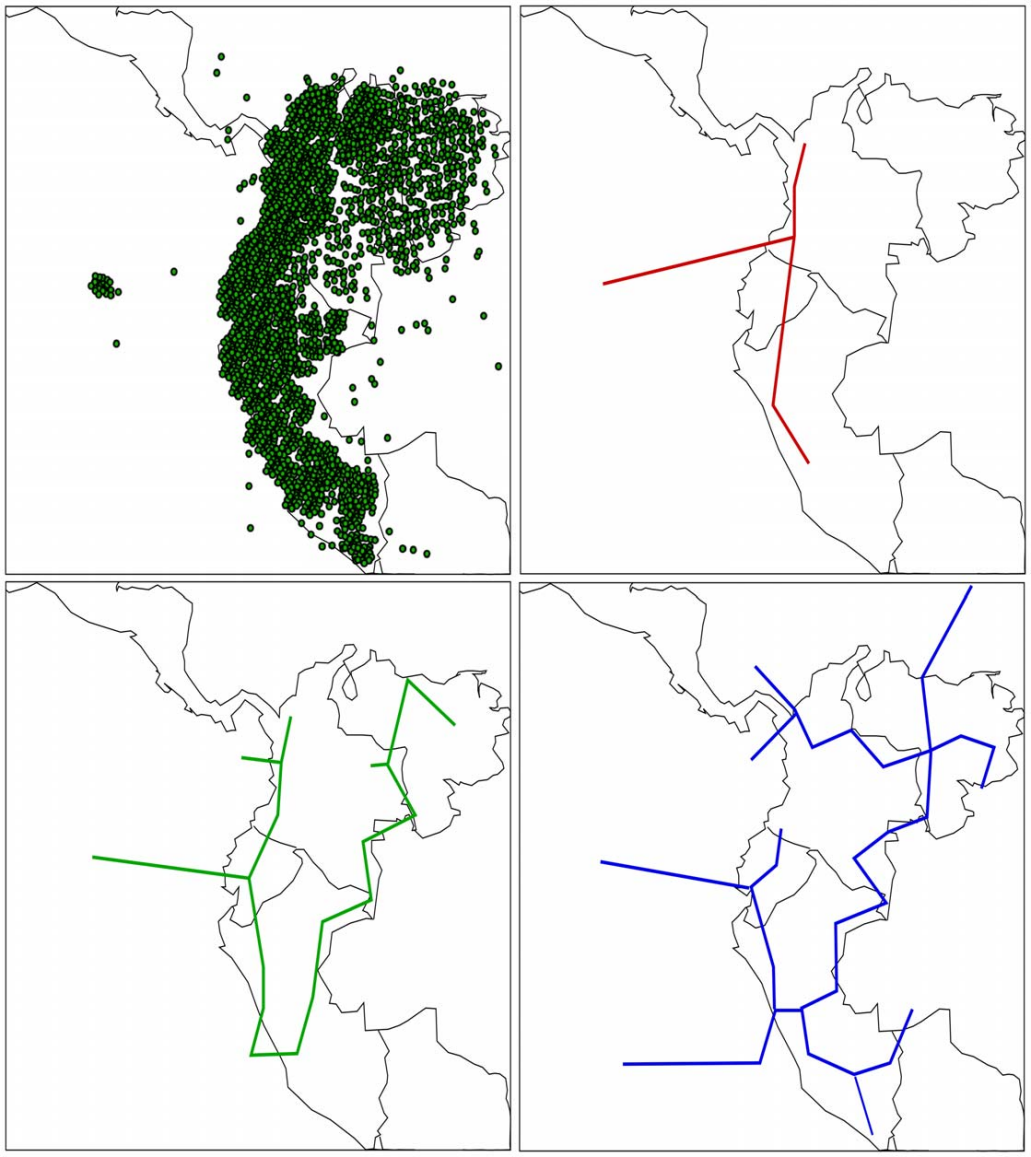
Panbiogeographical analysis from the Northern Andes. The 100,118 georeferenced localities of 1031 genera of plants and animals distributed across the Northern Andes, and general patterns of distribution obtained with the following parameters: cut value = 2, lmin = 10, lmax = 12, lmax.line = 16, and min-SI = 0.6 in MartiTracks.

Depending on the parameters used, analyses of the Northern Andes data generated several patterns including 3 to 27 generalized tracks. Figure 8 shows the three general patterns found with one of the parameter sets evaluated. The analyses required 15 to 30 minutes. These results prove the outstanding ability of MartiTracks to reduce data complexity and to find common distribution patterns with large data sets within a reasonable processing time.

## Discussion

As the amount of geographical information rapidly grows, the necessity for bioinformatic tools, able to deal with this kind of data, has increased. For panbiogeographical analyses, MartiTracks is a feasible quantitative alternative to traditional track analysis (e.g. Manual reconstruction or Craw's compatibility track analysis). Consequently, the ambiguity and the subjective factor, produced when overcrowded geographical points are evaluated [26], [27], are eliminated from the analyses. Another significant advantage of MartiTracks is that the geometrical approach eliminates large amount of time needed for analyzing large data sets as shown in the Northern Andes analysis. Thus, a single computer could easily deal with data sets involving ten of thousands of geographical records. Finally, by setting different distance parameters, which define the level of congruence, the users can explore several levels of resolution for analyzing their data sets according to their requirements.

## Materials and Methods

MartiTracks was written in Freepascal language under the Unix Operative System, Linux - Ubuntu 10.04 64 bits. Compiled versions of the program for Windows and Linux platforms, along with the source code are freely available under a GNU General Public license GPL 2.1 at http://tux.uis.edu.co/labsist/martitracks and http://code.google.com/p/martitracks

**Availability and Requirements**

Project name: MartiTracks

Project home page:

http://tux.uis.edu.co/labsist/martitracks

http://code.google.com/p/martitracks

Bug tracking http://code.google.com/p/martitracks

Operating system(s): Platform independent

(but 64 bits OS is recommended for medium/large data sets)

Programming language: Freepascal

License: GNU GPL 2.1

Any restrictions to non-academics: none
